# Active Opto-Magnetic Biosensing with Silicon Microring Resonators

**DOI:** 10.3390/s22093292

**Published:** 2022-04-25

**Authors:** Piero Borga, Francesca Milesi, Nicola Peserico, Chiara Groppi, Francesco Damin, Laura Sola, Paola Piedimonte, Antonio Fincato, Marco Sampietro, Marcella Chiari, Andrea Melloni, Riccardo Bertacco

**Affiliations:** 1 Dipartimento di Fisica, Politecnico di Milano, Via G. Colombo 81, 20133 Milano, Italy; francesca.milesi@polimi.it (F.M.); chiara.groppi@polimi.it (C.G.); riccardo.bertacco@polimi.it (R.B.); 2 Dipartimento di Elettronica, Informazione e Bioingegneria, Politecnico di Milano, Via Ponzio, 34/5, 20133 Milano, Italy; nicola.peserico@polimi.it (N.P.); paola.piedimonte@polimi.it (P.P.); marco.sampietro@polimi.it (M.S.); andrea.melloni@polimi.it (A.M.); 3 Istituto di Scienze e Tecnologie Chimiche “Giulio Natta” SCITEC CNR, Via Mario Bianco 9, 20131 Milano, Italy; francesco.damin@scitec.cnr.it (F.D.); laura.sola@scitec.cnr.it (L.S.); marcella.chiari@scitec.cnr.it (M.C.); 4 STMicroelectronics s.r.l., 20864 Agrate Brianza, Italy; antonio.fincato@st.com

**Keywords:** integrated optics, optical biosensing, magnetic labelling, microring resonator, lab on chip

## Abstract

Integrated optical biosensors are gaining increasing attention for their exploitation in lab-on-chip platforms. The standard detection method is based on the measurement of the shift of some optical quantity induced by the immobilization of target molecules at the surface of an integrated optical element upon biomolecular recognition. However, this requires the acquisition of said quantity over the whole hybridization process, which can take hours, during which any external perturbation (e.g., temperature and mechanical instability) can seriously affect the measurement and contribute to a sizeable percentage of invalid tests. Here, we present a different assay concept, named Opto-Magnetic biosensing, allowing us to optically measure off-line (i.e., post hybridization) tiny variations of the effective refractive index seen by microring resonators upon immobilization of magnetic nanoparticles labelling target molecules. Bound magnetic nanoparticles are driven in oscillation by an external AC magnetic field and the corresponding modulation of the microring transfer function, due to the effective refractive index dependence on the position of the particles above the ring, is recorded using a lock-in technique. For a model system of DNA biomolecular recognition we reached a lowest detected concentration on the order of 10 pm, and data analysis shows an expected effective refractive index variation limit of detection of $7.5\times 10^{-9}$ RIU, in a measurement time of just a few seconds.

## 1. Introduction

In diagnostics and biosensing, the demand for new tools is increasing year by year. Selectivity, sensitivity, response time, stability, reliability and reproducibility are just some of the parameters the researchers are looking to improve when developing a new technology [1].

Over the past decades many different biosensing platforms based on light have been proposed [2,3], aiming to realize compact, integrated and low cost optical biosensors suitable for large volume production. Among the most interesting proposed technologies, Surface Plasmon Resonance (SPR) [4], Photonic Crystals (PC) [5] and Silicon Photonics (SiPh) sensors are worth mentioning.

SPR and Localized Surface Plasmon (LSP) are among the most sensitive platforms under development, with detection limits down to $10^{-7}$ Refractive Index Units (RIU) for bulk and 1 pg/mm${}^{2}$ for surface sensing [6] in a commercial application. Gold nanoparticles were used in [7] to detect down to 0.74 pm of cerebrospinal diseases’ biomarkers, and [8] detected down to 10 fm of oligonucleotides with gold–silver nanostructures enhancing the Raman emission.

PC are gaining attention, with notable results introducing new detection techniques. Researchers detected avidin protein down to 15 nm or 1 μg/mL, with a surface mass density of 60 pg/mm${}^{2}$ corresponding to 100 ag [9] and a user friendly device based on smartphones detected influenza A H1N1 virus down to 138 pg/mL [10].

Exploiting the knowledge coming from telecommunication field and the improving quality in SiPh foundries [11], researchers have developed promising photonic integrated circuits (PICs) biosensors based on Mach–Zehnder interferometers (MZI) [12], resonating cavities [13,14], Bragg gratings [15] and Raman scattering [16,17].

MZI in particular are very promising solutions, with proven bulk sensitivities up to 20 μm/RIU and 10^−7^ RIU detection limit [18]. Researchers with a Young interferometer coupled to a CCD camera and a fast Fourier transform algorithm detected $9\times 10^{-9}$ RIU variation and down to 0.013 pg/mm^2^ for IgG antibodies with protein G [19].

Resonating cavities biosensors exploit the high Q-factor achievable to detect very low concentrations of analyte, down to single molecules. Spherical and thoroidal reached detection limits in the order of 10^−6^ RIU and sensitivity of 850 nm/RIU [20], while [21] reported a frequency-locked measurement method on a microtoroid able to detect fm shifts. MicroRing Resonators (MRR) have been proposed for many platforms and in a large variety of configurations. Cascaded MRR in the Vernier scheme [22] reached 24,300 nm/RIU, while [23] obtained a bulk sensitivity 912 nm/RIU with slot waveguide MRR.

In parallel to optical solutions, we assisted a rapid growth of magnetic biosensors [24,25], exploiting magnetic particles as molecular labels. The most studied platforms in the field exploit physical effects such as Giant Magnetic Resistance (GMR) [26,27], the Hall effect [28] and Magnetic Tunnelling for sensing magnetic labels upon biorecognition [29].

The sensing principle is based on the detection of a phase shift or absorption induced by the biological bindings of the analytes on the sensor surface. Both label-free and labelled approaches can be used with PICs. While un-labelled methods, in which just the analytes are the source of the effect on the sensor, usually allow a simpler sample preparation, the use of labelling techniques can improve performances in terms of sensitivity and speed. Among the various particles that can be used as a label, Magnetic NanoParticles (MNP) [30,31] can be exploited for biorecognition, magnetic sorting in sample preparation and as magnetic labels in surface free and surface based assays. In some works, external fields have been used to perform magnetic actuation during the assay [32,33] to attract labelled target molecules towards probes immobilized on the sensor surface or to activate the MNP motion and optically detect the presence of bound target molecules which alter relaxation times in surface-free assays [34,35,36]. Further, in surface-based assays, MNP can be used to produce a great enhancement of the signal when immobilized on the sensing surface. This has been demonstrated in SPR [37,38] with a sensitivity improved by a factor of four and in integrated MRR [39], with a biding step time lowered by a factor of 11 and a detection limit lowering from 124 pg/mL down to 57 pg/mL.

In this work we present a novel biosensing technique, named the Opto-Magnetic technique, which combines SiPh microring resonators and magnetic nanoparticles on a PIC. With the use of oscillating magnetic field and lock-in detection of the phase variation induced by the motion of immobilized MNP we show that quantitative analyte measurements on biomolecules such as DNA can be performed in a few minutes after biomolecular recognition, thus avoiding long measurements usually carried out in label-free optical platforms where the signal proportional to the analyte concentration is the difference of the sensor response after and before hybridization. In our approach, the presence of MNP labelling the target molecules on top of a surface specifically functionalized is detected after hybridization, thus solving typical issues of signal stability and parasitism affecting conventional measurements in which the signal must be continuously recorded over the hybridization time, on the order of hours.

In Section 2, the basic concept of the Opto-Magnetic technique is described, with deeper insights of the chip and the labelling in Section 3. The waveguide exposure and the functionalization step are reported in Section 4, while the experimental setup is described in detail in Section 5. Measurement procedures and collected results are in Section 6 and the final discussion and conclusions can be found in Section 7.

## 2. Opto-Magnetic Assay Concept

Integrated optical biosensors are based on the perturbation that the analytes induce on the light propagating in the waveguides. The surface of the waveguide core is in direct contact with the fluid containing the analyte that perturbs the exponential evanescent electromagnetic field extending outside the core. The target molecules immobilized on the waveguide surface therefore induce a variation of the phase of the optical mode proportional to the difference of the refractive index of the bound molecules with respect to the refractive index of the fluid surrounding the waveguide. In general the induced attenuation variation is negligible. The phase variation has to be converted in intensity variation in order to be easily detected with photodiodes and hence the sensible waveguide must be inserted in an interferometer. The literature is rich with theoretical studies and experimental results on different kinds of interferometric structures such as Mach–Zehnder, ring resonators and Fabry–Perot cavities, Bragg gratings and more, realized on a plethora of technological platforms [12,13,14,15,40].

However, the binding process between the molecules and the probes on the waveguide surface occurs on a time scale of minutes or hours; meanwhile the output power can also change due to other effects such as temperature variation of the overall device, density and refractive index change of the fluid, mechanical stress, misalignment of the input–output coupling, acoustic and environmental noise and so on. If, on one side, the basic principle of a PIC biosensor is pretty simple, on the other side a reliable and quantitative data detection requires accurate overall control and stabilization that often impair the exploitation in real applications.

In this work we propose a label approach for PIC biosensing to circumvent most of the environmental sources of noise, drifts and the relative risk of false detection. The proposed technique consists of labeling the analyte with MNP, for which the diameter is of the order of 100 nm, that is, a fraction of the waveguide width, to increase the optical field perturbation each time a particle is trapped and immobilized on the waveguide surface. This has been proposed in past literature [39], also because a suitably oriented constant magnetic field attracts the MNPs towards the functionalised surface and accelerates the binding process.

Here, instead, a periodic time varying magnetic field is used to induce a coherent and synchronous oscillation of all the bound MNPs and, as explained in the next section, of the output power signal that can be advantageously detected with a locking technique. The magnetic field is produced by an electromagnet placed under the SiPh chip. The electromagnet is designed to produce a large magnetic field gradient in the proximity of the sensing elements and to provide an attractive force to any susceptible material nearby.

The key advantage of this interrogation method is twofold: first, it is not necessary to monitor the entire long biological binding process while trying to extract the effect of the analyte from all the other parasitic effects by comparing with control sensors, and, second, the sensor can be interrogated after the binding occurs in an extremely rapid way. In Section 6, we have detected the desired analytes in the time scale of few seconds on samples prepared even days before the test.

**Figure 1:**
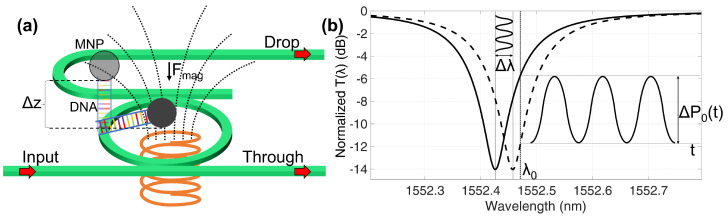
Opto−Magnetic dynamic labelling concept. (**a**) The MNP is subject to a magnetic force that produces a vertical dislocation of the biomolecule from the rest position; a varying external magnetic field causes the analyte oscillation. (**b**) The periodic phase perturbation shifts the transfer function T(λ), modulating the output light intensity $\Delta P_{o}$ when a laser has a fixed wavelength.

Figure 1 summarizes the concept of the interrogation technique. The probe molecules on the MRR surface trap the analyte with the linked magnetic bead. The time varying magnetic field induces an oscillation of the target-probe complex that shifts (Figure 1b) the ring resonant frequency and produces a variation of the detected optical power $\Delta P_{o}\left(t\right)$ at the output ports of the ring. A previous calibration of the sensor permits relates $\Delta P_{o}\left(t\right)$ to the number of immobilized MNPs and quantifies the target concentration.

## 3. Photonic Circuit and Magnetic Labelling

### 3.1. Optical Aspects

The variation $\Delta P_{o}$ of the power at the output of the interferometric photonic circuit, with respect to the input power $P_{i}$ can be written as:

(1) $$\frac{\Delta P_{o}}{P_{i}}=S_{s}\Delta_{env}=\frac{\delta T}{\delta \lambda }\frac{\delta \lambda }{\delta n_{eff}}\frac{\delta n_{eff}}{\delta_{env}}\Delta_{env}=S_{i}S_{\lambda }S_{env}\Delta_{env},$$

where $\Delta_{env}$ is the change of an environmental parameter in the surrounding of the waveguide and $S_{s}$ is the overall sensitivity of the biosensor to the environmental perturbation. In the typical biosensing approaches such environmental perturbation is the change of the refractive index of the fluid, of the bound molecules or of labels attached to the analytes. In the proposed technique, $\delta_{env}$ is the distance of the MNP from the waveguide. The effective index $n_{eff}$ of the waveguide mode is affected by the environmental parameter variation through the waveguide sensitivity $S_{env}=\delta n_{eff}/\delta_{env}$. The waveguide sensitivity $S_{env}$ is a major topic in the literature [1,2,15,41] and is investigated and quantified in this section for our case.

The effective index change $\delta n_{eff}$ induces a wavelength shift δλ of the transfer function of the interferometric structure [42,43] that does not depend on the structure itself and depends only on the group index $n_{g}$ of the waveguide,

(2) $$S_{\lambda }=\frac{\delta \lambda }{\delta n_{eff}}=\frac{\lambda }{n_{g}},$$

being $n_{g}=n_{eff}-\lambda \delta n_{eff}/\delta \lambda$. Finally, the output power variation depends on the derivative of the interferometric transfer function T(λ) with respect to the wavelength in the working point, named $S_{i}$.

From Equation (1) it is evident that a large biosensor sensitivity $S_{s}$ is achieved with a waveguide for which the effective refractive index is highly sensitive to the environmental parameter (large $S_{env}$), with a low group index and with a phase to amplitude converter—that is the interferometer—having a high spectral dependence (large $S_{i}$).

The choice of the waveguide technology and shape would permit us to optimize two sensitivity factors. $S_{\lambda }$ is the inverse of the group refractive index that can assume values from 1.5, for silica waveguides with low index contrast, to about 4.5 in the case of silicon photonic waveguides. $S_{\lambda }$ therefore plays a marginal role in the overall sensitivity $S_{s}$. For considerations of $S_{env}$, the reader can refer to the extensive literature. The choice of the waveguide technology however, is often related to other considerations such as reliability, suitability for volume production, footprint, cost, possibility of integration of active components as photodetectors and electronics [44] and so on. Silicon photonic technology is becoming easily accessible through commercial foundries that also offer MPW processes [11,45] and has been used for this work.

**Figure 2:**
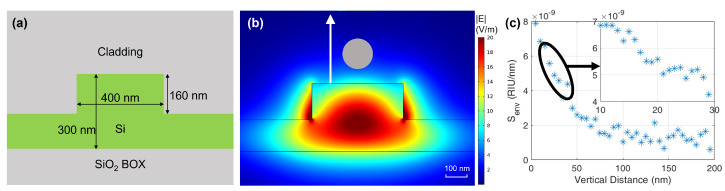
(**a**) Cross section and dimensions of the silicon photonic waveguide. (**b**) Transverse electric field distribution of the fundamental TE mode. (**c**) Environmental sensitivity $S_{env}$ extracted from numerical simulation for an MNP in the proximity of the waveguide. Inset: $S_{env}$ around 20nm MNP−waveguide distance.

Figure 2a shows the rib cross section of the silicon photonic waveguide used in this work; 160 nm high × 400 nm wide and buried in a silicon dioxide layer. The cladding is removed to expose the waveguide where needed, implementing the sensing element. Since the Opto-Magnetic technique exploits the perturbation caused by oscillating magnetic nanoparticles, numerical simulations have been used to evaluate the effect of their proximity, that is, $S_{env}$. In Figure 2b, the TE mode electric field distribution calculated with the COMSOL Multiphysics model is shown. The MNP is modelled as a 130 nm diameter sphere with an iron oxide inner core and a dextran-polimeric shell. Simulations have been carried out to calculate the variation of effective refractive index $\Delta n_{eff}$ as a function of the vertical distance of the MNP from the waveguide surface. From this information it is possible to estimate $S_{env}=\delta n_{eff}/\delta_{env}$, $\delta_{env}$ being the MNP-waveguide distance variation and the result is reported in Figure 2c. Considering a single MNP to be 20 nm above the MRR, the environmental sensitivity results in $S_{env}≃$ 5 × 10^−9^ RIU/nm.

A comprehensive analysis and optimal design of MRR and Mach–Zehnder interferometers for biosensing can be found in [41]. The choice of the interferometric structure and the working point in the spectral response defines the sensitivity $S_{i}$. It is worth noticing that, for values of practical interest, both structures can achieve the same $S_{i}$ if they have the same slope of the magnitude transfer function vs the wavelength. The maximum value of $S_{i}$ is limited by the attenuation of the waveguides and mainly by the electronic interrogation system, the laser and photodetector noise, the thermal stabilization, vibrations and other practical issues [41].

**Figure 3:**
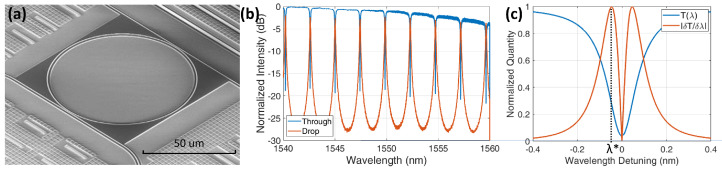
SiPh chip. (**a**) Picture of the MRR: waveguides for Input, Through and Drop are visible; (**b**) Through and Drop spectral response of an exposed MRR immersed in saline solution. (**c**) Modelled Through transfer function T(λ) and its slope $S_{i}\left(\lambda \right)=\delta T\left(\lambda \right)/\delta \lambda$.

In this work, a ring resonator is used mainly to reduce the footprint, design a compact multipoint biosensor and ease the microfluidic circuit. The platform used for the measurements is a 6×5 $mm^{2}$ SiPh microchip provided by STMicroelectronics. Each chip has 14 MRRs with a diameter of 80 μm in the Drop-Through configuration (see Figure 1a), each one with Ge photodiodes. Figure 3a shows a microphotograph of a sensing ring with the upper cladding removed to expose the MRR waveguide. The MRRs’ couplers gaps are designed to obtain four different values of Q-factor, from 5000 to 65,000. A group of four extra MRRs is placed on one side of the chip for the control and comparison. Being fabricated in a Multi-Project Wafer run, the waveguides lie under multiple alternated layers of silicon dioxide and silicon nitride, whose removal is described in the next Section. The measured transfer function at Through and Drop ports in saline solution after cladding removal are shown in Figure 3b. The Free Spectral range FSR of the ring is 2.5 nm around 1550 nm. The group refractive index $n_{g}$ can be retrieved from the FSR expression:

(3) $$FSR=\frac{\lambda^{2}}{n_{g}2\pi R}.$$

For a radius R= 40 μm and λ= 1560 nm, the group index $n_{g}=3.89$ is obtained. With these values, and through Equation (2), a sensitivity of $S_{\lambda }=\lambda /n_{g}≃$ 400 nm/RIU is estimated.

Finally, to evaluate $S_{s}$, the interferometric sensitivity $S_{i}$ has to be calculated. From Figure 3c, it is clear that the best working point, where $S_{i}$ is the maximum, is obtained at the maximum slope of the transfer function T(λ), so at the δT/δλ peak indicated as $\lambda^{*}$. $S_{i}$ being highly dependent on the waveguide exposure, on the quality of the MRR surface, on the functionalization coating layer and on the optical power in the cavity, T(λ) and δT/δλ have been obtained for each acquisition as explained in Section 6. We can anticipate that, according to our experimental setup, at the peak $\lambda^{*}$, the sensitivity $S_{i}$ is in the order of a few mV/pm. With this information, we can estimate the whole $S_{s}=S_{i}S_{\lambda }S_{env}$ to be ∼μV/nm, meaning that for a single MNP being displaced by 1 nm we should expect a ∼ 1 μV change in the sensor output.

The sensitivity of an optical biosensor sometimes indicates the so called bulk sensitivity $S_{b}$, which is a measurable value that quantifies the wavelength shift (usually expressed in nm) with respect to the change of refractive index in the cladding, such as a changing in the fluid under test. Collecting samples with known index solutions [46,47], our chips showed $S_{b}=$ 20 nm/RIU, comparable with other rib waveguides in high index contrast platforms.

### 3.2. Magnetic Aspects

The magnetic nanoparticles used in this work are ensembles of superparamagnetic particles grouped together by a polymer matrix and functionalized on the surface. Magnetic objects are defined as superparamagnetic when they are smaller than a critical dimension, typically in the order of a few nm for magnetite $Fe_{3}O_{4}$ or magnemite $Fe_{2}O_{3}$, causing them to be in a single domain configuration. The magnetization can then rapidly jump between the two possible states (parallel and anti-parallel to the anysotropy axis), leaving no magnetic remanence and thus behaving as paramagnetic, but with a higher relative susceptibility and with the single atomic moments keeping their ferromagnetic order. The assembled MNP presents a magnetic moment that is the vectorial sum of all the superparamagnetic ones and a diameter that usually goes from tens of nanometers to a few microns [48,49].

As schematically shown in Figure 1a, by applying a magnetic field, the MNP is subjected to a force that, in a point-like approximation, can be written as [50]:

(4) $$F_{\mathrm{mag}}=\frac{1}{2}\mu_{0}V_{b}\Delta \chi \nabla H^{2}\left(r_{c}\right),$$

where $\mu_{0}=4\pi \;\cdot \;10^{-7}$ H/m is the permeability of free space, $V_{b}$ is the volume of the MNP, $\Delta \chi =\chi_{b}-\chi_{fluid}$ is the difference between the magnetic susceptibility of the particle and the surrounding medium and $H(r_{c})$ is the external magnetic field at the MNP center $r_{c}$.

The force is directed towards the electromagnet placed under the chip for both positive and negative values of the magnetic field, attracting the MNP towards the chip surface. For a sinusoidally varying magnetic field at frequency $\omega_{0}$, the magnitude is $H{\left(t\right)}^{2}$, as its gradient is $\nabla (H{\left(t\right)}^{2})$ and hence the force $F_{\mathrm{mag}}$ (4) oscillates at $2\omega_{0}$, being:

(5) $$H^{2}\left(r,t\right)={\left|H_{0}\left(r\right)\right|}^{2}\sin^{2}\omega_{0}t=\frac{|H_{0}{\left(r\right)|}^{2}}{2}\left(1-\cos2\omega_{0}t\right).$$

The wavelength shift Δλ changes at the rate $2\omega_{0}$ as well as, in the small signal regime, the optical intensity (1), these two being related by the sensitivity $S_{i}=\delta T\left(\lambda \right)/\delta \lambda$, that is, the slope of the MRR transfer function T(λ),

(6) $$\Delta P_{o}\left(\lambda_{0}\right)=S_{i}\left(\lambda_{0}\right)\Delta \lambda_{2\omega_{0}},$$

where $\Delta \lambda_{2\omega_{0}}$ is the amplitude of the oscillation of the resonating frequency around $\lambda_{0}$ induced by the oscillation of the MNP. Using a Lock-In Amplifier (LIA), it is possible to extract the intensity of the oscillation at $2\omega_{0}$ using the sinusoidal signal driving the electromagnet as a reference to a second-harmonic demodulator and hence $\Delta \lambda_{2\omega_{0}}$.

## 4. Chip Preparation

### 4.1. Silicon Photonic Chip Preparation

The Silicon Photonic chip has been realized with an MPW run in STm. Waveguides are buried in a multilayer structure of multiple alternated silicon dioxide ($SiO_{2}$) and silicon nitride (SiN) layers 5.3 μm thick [51]. In order to expose the optical waveguides, it is necessary to remove these layers by means of dry and wet etching through Chromium (Cr) hard masks fabricated by optical lithography, sputtering and lift-off.

To fabricate the hard mask, a 1 μm thick layer of AZ5214E photoresist is spin-coated over the chip surface. Then the patterning is obtained by a positive-exposure optical lithography and development procedure, leaving hardened photoresist islands over the MRR areas. A 150 nm layer of Cr is deposited all over the surface by the sputtering technique and the following acetone lift-off creates the Cr hard mask with apertures to the MRR.

Two different RIE processes have been optimized to remove the upper cladding structure of $SiO_{2}$ and SiN. The first one is a Bosch-like process where two gases are sequentially alternated inside the chamber: $SF_{6}$ for etching (step duration 7 s) and $C_{4}F_{8}$ for side walls passivation ( 5 s). Due to the time required to pump-away the reactive species inside the chamber, intermediate pumping steps ( 5 s) between gas injections are added to avoid a mixture of $SF_{6}$ and $C_{4}F_{8}$ in the vacuum chamber that can negatively affect the etching profiles and rate. The Bosch-like cycle parameters are listed in Table 1 and the sequence has to be repeated until the silicon waveguide core is reached. Finally the chips are processed with wet etching, immersing them into an HF solution for 3 min in order to remove all the $SiO_{2}$ surrounding the sides of the waveguides.

### 4.2. Chip Functionalization

To bind the single strand DNA (ssDNA) probes, the chips were functionalized at the SCITEC-CNR laboratories, with the polymer MCP-4, copoly(DMA-NAS-MAPS) [52,53,54], obtained by Lucidant Polymers Inc. (Sunnyvale, CA, USA). MCP-4 is a ter-polymer made of N,N-dimethylacrylamide (DMA), N,N-acryloyloxysuccinimide (NAS), and 3-(trimethoxysilyl) propyl methacrylate (MAPS). The three components have different functions: the DMA is the backbone providing surface binding, NAS is the reactive ingredient able to bind to the amino groups present in the ssDNA probes and MAPS stabilizes the coating. The goal is to bind molecules in an active conformation so that they preserve their functional activity.

The surface was first cleaned with $O_{2}$-Plasma for 15 min. The chip was then immersed in a MCP-4 solution, which is 1% *w*/*v* in water solution of ammonium sulfate at 20% saturation, for 30 min. After a DI water rinse and $N_{2}$ dry step, they sit at 80 ${}^{\circ }C$ for 15 min. The ssDNA probes were dissolved in the printing buffer ( 150 mm sodium phosphate pH 8.5, 0.01% Sucrose monolaurate) to a concentration of 10 μm and printed by a piezoelectric spotter, SciFLEX ARRAYER S12 (Scienion, Berlin, Germany), onto the coated chip. After the spotting step, the chip was incubated overnight, and all residual reactive groups of the coating polymer were blocked by dipping the chip in a blocking solution ( 50 mm ethanolamine, 0.1 m Tris, pH 9.0) for 60 min. The ssDNA sequences are purchased by Metabion International AG and are shown in Table 2. With a base pair length of 0.34 nm [55], 60 base pair sequences are expected to be 20 nm long when in a double strand structure. As a reference, some MRRs are left unspotted.

### 4.3. DNA Hybridization

The hybridization process is implemented in the microfluidic cell in which the chip is embedded. First, the ssDNA probe immobilized on the microchip is hybridized with the complementary biotinylated ssDNA target diluted at different concentrations in a saline sodium citrate solution (SSC).

The second step binds the magnetic nanoparticles. The used beads have a diameter of 130 nm, consisting of streptavidin coated NanoMag®-D from Micromod Partikeltechnologie GmbH, which do not display a sizeable sedimentation due to Brownian motion. MNP are diluted in Phosphate Buffered Saline (PBS) solution: the concentration has been chosen to have an overabundance of labels, in order to force the surface-bound targets to be the limiting factor in the biochemical recognition reaction. With a repeated wavelength scan and an algorithm to find the resonance it is possible to follow the molecular recognition process in real-time, even if not necessary for the scope of this work, as the Opto-Magnetic measurements are performed after the hybridization. On the other hand, this corresponds to the standard procedure for optical biosensing with MRR, so that it can provide a sort of benchmark for the Opto-Magnetic approach.

The needed solutions are listed in Table 3. These are preloaded in the input pipe connected to the microfluidic cell adding a small air bubble (≃ 10 μL) at every liquid interface to avoid mixing. As soon as the first solution (washing) wets the chip surface, the measurement starts. After a few minutes to measure the baseline, the hybridization protocol (reported in Table 4) begins. The incubation solution containing the complementary DNA is introduced into the fluidic cell. To avoid target depletion, a slow continuous flux is maintained.

The dsDNA forms fast: in 10 min the label free hybridization is mostly completed and after 30 min the next step starts. A washing solution is then used to gently remove the unbound molecules. After that, the buffer is fluxed on the chip for a few minutes to create a baseline for the labelled hybridization and then the functionalized MNP solution is introduced into the cell, with flux-pause steps to help molecular recombination. Due to the high biotin-streptavidin affinity and the larger impact of magnetic beads on the evanescent field as compared to small DNA molecules, the resonating wavelength rapidly shifts for the sensing MRR. After 60 min, the difference signal is almost saturated and the buffer solution is used to clean the surface from the unbound beads.

All the solutions, after passing through the fluidic cell, enter the syringe where they are mixed together and so considered waste. However, a different type of fluidic system can keep the fluids separated to be used again, maybe by putting in a series of other cells, yet a small loss in efficiency shall be expected in the steps following the first hybridization due to analyte depletion and partial cross-contamination.

## 5. Experimental Setup

**Figure 4:**
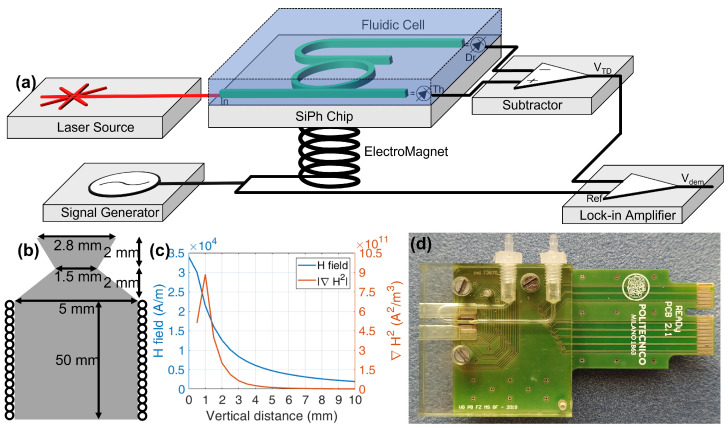
Opto−Magnetic platform. (**a**) Conceptual setup scheme: tunable laser source with drivers, integrated photonic chip in the microfluidic cell, LIA, signal generator with electromagnet; (**b**) Electromagnet geometry and (**c**) experimental magnetic field characterization; (**d**) photo of the Printed Circuit Board with SiPh chip and the assembled fluidic cell assembled.

The experimental setup is illustrated in Figure 4a. The laser wavelength is set by tuning the diode temperature and current and is vertically coupled to the chip through an optical fiber and a grating coupler on chip. The photonic chip has several MRRs each with two output ports named Through and Drop with integrated Germanium photodiodes and a transimpedance amplifier that detect the output optical signals. The signals are demodulated by a Lock-in Amplifier (LIA) and subtracted to remove the common mode term. A sinusoidal signal generator drives, at the desired intensity and frequency, the electromagnet for the generation of the magnetic field. The same signal is used as a reference for the demodulation by the LIA. The whole setup is controlled by a PC, setting the laser wavelength and acquiring the signals. The fluidic system is composed of a programmable syringe pump and a fluidic cell to put the liquid in contact with the sensors’ area.

The laser temperature driver is a Temperature Controller TED200, while the current driver is a Laser Diode Controller LDC210. The laser source is a commercial DFB diode laser from JDS Uniphase for telecom applications with 700 kHz spectral linewidth. The coefficients for wavelength tunability around 1560 nm are 1.3 pm/mA at 25 °C for current and 93 pm/°C at 200 mA for temperature.

The laser output is coupled to a Single Mode Fiber (SMF) and then to an SM 1 × 4 PLC Splitter. The four output SMFs are connected to a custom made 8-channels Fiber Array interposer from W2 Optronics to inject light into the photonic chip through grating couplers. The precise alignment is obtained with a 3-axis XYZ micropositioner and an ad-hoc 3D-printed holder for the fiber array.

The photonic chip is mounted on a custom made Printed Circuit Board (PCB) specifically designed to hold the chip and the fluidic cell and the photodiodes are wire-bonded to the PCB pads. A flat cable with an edge connector brings the signal to the amplification stage, designed and fabricated by Elite srl, Italy. The photodiodes are reverse biased at $V_{R}=$ 1.225 V. A low noise Trans Impedance Amplifier (TIA), with gain set by a 20 kΩ resistor, converts the photocurrents to voltages between 1.225 V and 3.3 V. Through and Drop voltages are read in differential modes, leading to $V_{TD}=V_{Through}-V_{Drop}$. The $V_{TD}$ signals, one for each connected MRR, are read by the PC via the Data AcQuisition (DAQ) board and by the LIA (HF2LI from Zurich Instrument Ltd). The $V_{TD}$ signal is demodulated by the second harmonic of the reference frequency with a 5 Hz bandwidth filter. The demodulated signal $V_{dem}$ at the LIA output is read by the PC DAQ, a PCI-6035E board with a BNC-2120 interface from National Instruments.

The internal signal generator of the LIA generates the sine wave signal with frequency $f_{EM}$ and peak $V_{EM}$. The signal controls the power supply BOP-36-12M from Kepco Inc. (Flushing, NY, USA) that drives the electromagnet, shown in Figure 4b. The electromagnet has an iron core 50 mm long with a 5 mm diameter. The top extremity of the core has a bottle-neck shape, in order to maximize $\nabla H^{2}$. Sixty-two turns of 0.8 mm diameter copper wire are tightly winded around the core. The coil shows an electric resistance of 1 Ω. In this configuration with a current $I_{EM}=$ 2 A it is possible to obtain a gradient in the range $\nabla H^{2}=10^{11}÷10^{12}$$A^{2}/m^{3}$ 1 mm from the electromagnet’s top surface, as shown in Figure 4c.

A custom LabView software controls the temperature laser driver to obtain a temperature ramp to scan the wavelength across an FSR of the MRR while acquiring both $V_{TD}$ and $V_{dem}$.

The fluidic system uses the syringe pump AL-1000 from WPI Ltd, manually operated in aspiration mode, with a 15 mL volume syringe. The PMMA cell (Figure 4d), designed by HTA srl (Italy), brings the liquid in contact with the chip surface thanks to a 1×1 $mm^{2}$ cross section channel above the MRRs. Sealing is assured by a silicone O-ring. Two slits are milled in the cell to allow the fiber array to reach the optical chip. Holes are present to align and tightly fix the fluidic cell to the PCB. A 1.5 mm inner diameter, 1 m long silicone pipe connects the syringe to the fluidic cell and it is used to sequentially preload all the needed solutions.

## 6. Measurements and Results

### 6.1. Hybridization

**Figure 5:**
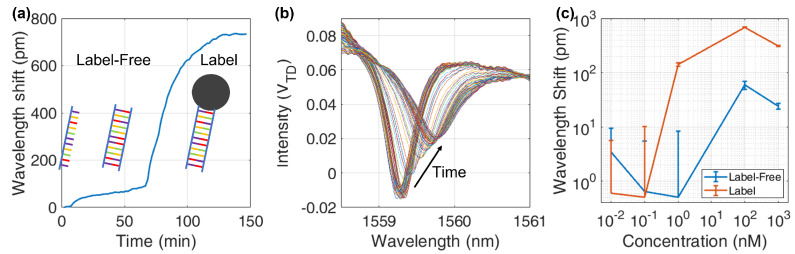
Measured effects of the hybridization: (**a**) Resonance shift difference between functionalized and control MRRs in case of funtionalized, unlabel and labelled detection ( 100 nm concentration). (**b**) Collection of MRR spectral responses acquired from the sensing MRR at 100nm during the binding: the ring resonance shifts and the Q−factor reduces. (**c**) Wavelength shifts at different concentrations for label−free and labelled bindings. Labelled shifts are at least ten times larger than label−free.

The hybridization processes, dsDNA formation and MNP binding, produce a resonance shift of the functionalized ring with respect to the control ring that undergoes only common fluctuations. The difference between the two acquired shifts represents the molecular recognition signal, while thermal fluctuations and non-specific binding are cancelled by the subtraction. An example of differential acquisition is shown in Figure 5a for a 100 nm concentration. Besides shifting the resonance, the presence of MNPs increases the roughness of the waveguide surface and hence the attenuation and scattering of the light field. The main affected parameters of the ring spectral response are the quality factor *Q* of the resonance and the Extinction Ratio, both decreasing as more beads attach to the surface, as shown in Figure 5b.

The resonance detection algorithm performs a Lorentzian curve fit to the data to find the resonating wavelength of each MRR. The difference between sensing and control ring responses is evaluated at the end of both processes, considering 5 min of plateau data before and after the process. Average shifts are plotted in Figure 5c; the error bar of each point is evaluated as the quadratic sum of the standard deviation in the plateau before and after hybridization. It is clear that the uncertainty limits the label-free detection. At 100 nm concentration, sensing and control MRR show a different response, while at 1 nm they are no more distinguishable, meaning that the Lowest Detectable Concentration (LDC) has been reached and it lays in the range 1–100 nm. The same effect is visible for labelled measurements, but in this case sensing and control MRR show a comparable shift at lower concentrations. The shift enhancement produced by the MNP is large enough to push the LDC down by at least one order of magnitude of target concentration, in the range 0.1–1 nm.

The common definition of Limit of Detection LoD for this kind of system is three times the standard deviation of the measured effective refractive index $\sigma_{n_{eff}}$ [41], such that, using Equation (2),

(7) $$LoD=3\sigma_{n_{eff}}=3\frac{n_{g}}{\lambda }\sigma_{\lambda },$$

we have a relation between the uncertainty in the retrieved wavelength $\sigma_{\lambda }$ and the LoD. In this case, with $\sigma_{\lambda }∼$ 10 pm we obtain $LoD∼7.5\times 10^{-5}$ RIU.

This analysis clearly shows the weak points of the conventional technique for optical biosensing. First, high stability over hours is needed to measure small variations of resonance wavelength disentangling them from spurious effects due to thermal, optical or mechanical drifts. Second, the optimized biological process of molecular recognition seldom requires conditions that are not easily compatible with the optical tracking method such as sample shaking or stirring and temperature and humidity values inside specific ranges. The Opto-Magnetic technique, instead, interrogates the system after the biomolecular recombination, allowing the binding to be performed in the required conditions to optimize the recognition efficiency, a dominant factor when dealing with low analyte concentrations.

### 6.2. Opto-Magnetic Measurements

The Opto-Magnetic technique is now presented and discussed. As soon as the hybridization process is over, the chip is kept in a PBS bath and the chip can be interrogated even after several days.

An oscillating magnetic field at frequency $f_{EM}$ is applied below the MRRs forcing the MNPs to oscillate. As explained above, this modulates the resonating wavelength and produces a light intensity modulation at $2f_{EM}$ on the photodiodes. The photodiodes’ signals $V_{TD}$ are sent to the LIA where a demodulator with a 5 Hz bandwidth filter extracts the second harmonics of the reference frequency $f_{EM}$ retrieving the amplitude $V_{dem}$ of the photodiode signal oscillation. The software drives the laser temperature controller to produce a wavelength ramp, scanning over a whole FSR with 1 pm steps at a 10 pm/s rate.

The DAQ acquires the second harmonic signal $V_{dem}$ and the photodiodes’ intensity $V_{TD}$, which is used to calculate the derivative with respect to wavelength $dV_{TD}/d\lambda$. The second harmonic $V_{dem}$ and the intensity-derivative are used to calculate the actual wavelength shift rearranging Equation (6):

(8) $$\Delta \lambda_{2\omega_{0}}=\frac{\Delta P_{0}\left(\lambda_{0}\right)}{S_{i}\left(\lambda_{0}\right)}\;↔\;\Delta \lambda^{*}={\left(\frac{V_{dem}}{dV_{TD}/d\lambda }\right)}_{\lambda^{*}}.$$

Scan and calculation are repeated for both functionalized and reference MRR, and are performed for different concentrations of the target solutions.

### 6.3. Opto-Magnetic Results

**Figure 6:**
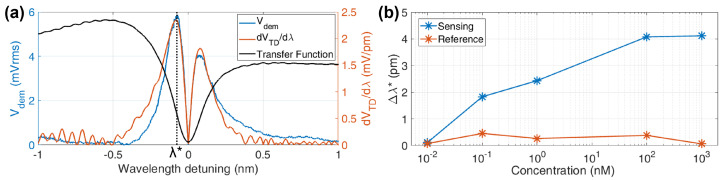
Opto−Magnetic measurements and results. (**a**) Typical acquired curves: the MRR transfer function as $V_{TD}$ intensity (no scale), its derivative absolute value which superimposes to the second harmonics $V_{dem}$. (**b**) Resonance shift dependence on the target solution concentration from 10pm to 1μm.

The Opto-Magnetic measurements are now presented, with the collection of acquisition showed in Figure 6a.

From the spectrum intensity, $V_{TD}$, its derivative, is calculated and the second harmonics demodulated signal $V_{dem}$ is extracted from the LIA. The shape of the $V_{dem}$ curve is proportional to the $V_{TD}$ derivative (see Equation (6) and Figure 1b), producing two peaks around the resonance wavelength. The peak positions $\lambda^{*}$, being in correspondence with the maximum slope of the transfer function, allows a low noise evaluation for both $V_{dem}$ and $dV_{TD}/d\lambda$. The resonance oscillating amplitude $\Delta \lambda^{*}$ is then obtained, dividing the highest peak values of the two quantities.

The shifts for the sensing and reference MRRs with the tested DNA concentrations are visible in Figure 6b, where one sample for each concentration was tested. On the control MRR, the effect of oscillating MNP is similar for all the concentrations, while it is clearly visibly a direct relation in the DNA functionalized ones: the more complementary DNA is present during the hybridization, the more MNP are attached to the chip surface and the more intense an oscillating signal is produced by the ring. As for order of magnitudes, the technique is distinguishing pm-range shifts over $\lambda_{0}=$ 1.56 μm laser wavelength. Considering the reference samples, the average and the standard deviation are $\mu_{ref}=$ 250 fm and $\sigma_{ref}=$ 175 fm, indicating that, with this chip, the Limit of Detection LoD can be estimated to be in the range 10–100 pm, or by using Equation (7), $LoD_{scan}≃1.3\times 10^{-6}\;RIU$, a strong improvement from the previous value.

**Figure 7:**
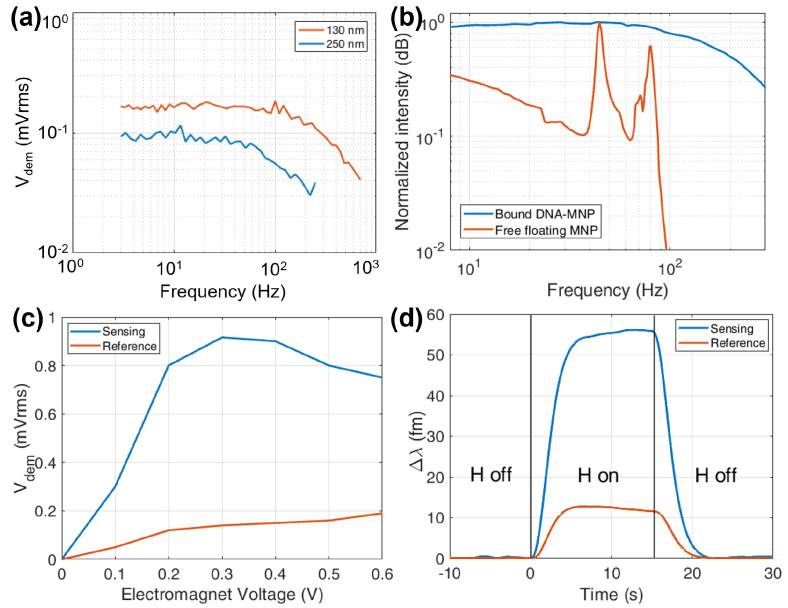
Calibration of the Opto−Magnetic measurements parameters and high precision detection. (**a**) Response comparison between different MNP sizes, the higher mobility favours smaller particles. (**b**) Frequency response comparison for MRR with bound and free MNP, each configuration normalized to its sensing’s maximum. (**c**) Electromagnet voltage calibration to find the optimum signal. (**d**) On−Off Opto−Magnetic signal at 10pm concentration with 0.1 Hz bandwidth filter: fast and low−noise measurement.

The MNP dimension affects the intensity of the signal. An example is plotted in Figure 7a that shows 130 nm diameter particles compared with 250 nm diameter ones. The magnetic force (Equation (4)) is proportional to the MNP volume and $F_{mag}∼R_{MNP}^{3}$ increases the opto-magnetic effect. In contrast, the streptavidin binding sites are on the MNP surface ($∼R_{MNP}^{2}$) and a higher number of receptors increases the number of molecules fixing the particle to the chip surface thus reducing its range of motion while on the unbound MNP surface the attracted DNA cause the hydrodynamic volume $V_{h}$ of the particle to increase, reducing the overall mobility. There is therefore a trade-off value and in our tests the 130 nm diameter turned out to be the optimal commercial solution.

To properly put in motion the MNP and maximize the oscillation swing, the magnetic field has to oscillate at the right frequency. The correct range should be high enough to avoid 1/f noise and lower than a possible pole in the DNA–MNP dynamic system. Fixing the wavelength at the maximum slope of the MRR transfer function and sweeping the magnetic field frequency $f_{EM}$ it is possible to see, reported in Figure 7b, that for properly bound DNA–MNP, the frequency response is flat until 100 Hz, where it starts to drop. The behaviour recalls an overdamped system, in which $F_{mag}$ is the external force, the DNA molecules act as springs with their own stiffness and the fluid produces drag. In the case of free-floating MNP, i.e., not bound to the biomolecule, several resonance behaviours arise for both sensing and reference MRR. Spurious resonances are most probably caused by rotational movements of unbound MNP, under the effect of the AC magnetic field and the fluid drag. Specifically, the Brownian relaxation frequency for suspended particles is:

(9) $$f_{B}=\frac{k_{B}T}{6\pi \eta_{fluid}V_{h}},$$

where $k_{B}$ is the Boltzmann constant, *T* the temperature, $\eta_{fluid}$ the viscosity of the fluid and $V_{h}$ the hydrodynamic volume of the MNP. At ambient temperatures, considering a PBS viscosity $\eta_{fluid}≃$ 1 mPas [56] and a hydrodynamic radius of 65 nm, the Brownian relaxation frequency is about 190 Hz. Since the system extracts the $f_{EM}$ second harmonic, the rotation’s effects are expected at 95 Hz in fair agreement with the second peak in Figure 7b. The first peak, at a lower frequency (∼40 Hz), can be due to particles interacting with substrate. After a proper rinse process, however, these peaks are suppressed and the optimal frequency to drive the electromagnet is chosen to be $f_{EM}=$ 80 Hz, where the signal to noise ratio is maximized.

With a similar process, the correct intensity of the magnetic field has been found sweeping the voltage $V_{EM}$ applied to the driver of the electromagnet in order to maximize $V_{dem}$. In Figure 7c, a wide maximum between 0.2 V and 0.5 V (corresponding to 2 ÷ $5\times 10^{3}$ A/m peak values for *H* on the chip) for the functionalized sensor is visible, while the reference shows a steady increase mostly due to electromagnetic induction in the nearby readout electronics. Measurements were conducted at $V_{EM}=$ 0.5 V peak voltage.

At the lowest tested concentration of 10 pm, the sensing and reference signals in Figure 6b are hardly distinguishable, so the LDC is reached. To improve the signal to noise ratio, one should decrease the LIA bandwidth, but this would imply a hardly acceptable decrease of the wavelength scan speed, causing the acquisition time to increase.

To overcome this difficulty we developed a second detection method at a fixed wavelength. First, a scan is used to evaluate the transfer function slope, then the laser wavelength is tuned on the position of maximum derivative, the LIA filter is set to a narrower bandwidth of 0.1 Hz and the AC magnetic field is temporarily switched on for a few seconds and then off before the electromagnet heats the chip. So the interrogation system is being activated for just the time needed to acquire some seconds of light modulation. Following this procedure, it is possible to produce a low-noise On-Off Opto-Magnetic measurement that lasts long enough to be accurately filtered, as shown in Figure 7d. Evaluating a 10 s time frame at the plateau, the reference MRR response presents a mean $\mu_{ref}=$ 12.3 fm with an error $\sigma_{ref}=$ 0.35 fm while the sensing MRR $\mu_{sens}=$ 55.5 fm and $\sigma_{sens}=$ 0.6 fm. The not zero $\mu_{ref}$ is most probably caused by a few unbound MNP still floating close to the MRR and some electromagnetic induction collected by the system closer to the electromagnet. The comparison between the two signals $\left(\mu_{sens}-\mu_{ref}\right)>3\left(\sigma_{sens}+\sigma_{ref}\right)$ suggests that this technique may push the lowest detectable concentration even lower than the concentration limit of 10 pm tested so far and considering a $\sigma_{\lambda }≃$ 1 fm and applying Equation (7) we obtain $LoD_{on-off}≃7.5\times 10^{-9}$ RIU.

This is a remarkable result: obtained with a measurement time in the order of just few seconds, better than any other rib silicon waveguide-based MRR platforms and comparable with the state of the art in optical biosensing [57,58]. So, considering that our bulk sensitivity is calculated in $S_{b}≃$ 20 nm/RIU and other platforms can reach values hundreds times larger we can expect great improvement in performances just by applying the Opto-Magnetic technique to other high-sensitivity platforms.

## 7. Conclusions

To conclude, in this work we presented a novel technique, called Opto-Magnetic, to perform actively-labelled biosensing with a silicon photonics ring-resonators’ based platform. The labels are magnetic nanoparticles, which can be actuated with the use of an external magnetic field. An oscillating field produces a shift of the resonance condition of the cavity that causes a variable optical output intensity. The frequency component associated with the magnetic actuation can be extracted with a locking demodulator. For a model system of DNA recognition, we tested successfully down to 10 pm concentration, extracting a theoretical limit of detection $LoD_{on-off}∼7.5\times 10^{-9}$ RIU, comparable to the state-of-the-art solutions in optical biosensing.

It is worth noting that the time needed to measure the target concentration goes from a few minutes for the whole FSR scan, to just a few seconds for the On-Off mode, as the readout can be performed after the hybridization protocol. This approach can be used for each kind of target molecule (nucleic acids, antibodies, proteins, etc.) provided that a suitable step for magnetic labelling is added to the bioassay. Elisa-like protocols being widely used in biological applications, several steps in molecular binding are easily accessible for a large variety of biomolecules. The interrogation being performed after the molecular binding, the biorecognition step that usually lasts up to several hours, with our technique can be performed without worrying about measuring the resonance condition, thus it can be accomplished with temperature, humidity and mechanical agitation parameters optimized for the specific analyte. The Opto-Magnetic technique improves the performance, robustness, speed and reliability of existing refractive index-based integrated photonics biosensing platforms.

## 8. Patents

“Opto-Magnetic sensor device and molecular recognition system” (http://hdl.handle.net/11311/1126863 accessed on 29 March 2022).

## Figures and Tables

**Table 1:** Reactive Ion Etching Bosch like process parameters: for each step, duration time, gas fluxes, RF and Inductively Coupled Plasma power and sample potential are reported.

	Time	$SF_{6}$	$C_{4}F_{8}$	RF	ICP	Pressure	DC
	s	sccm	sccm	W	W	mBar	V
Clamp	20	/	/	/	/	/	/
Cooling	5	/	/	/	/	5	/
**Repeat**							
Stabilization 1	5	80	/	/	/	5	/
Etching	12	80	/	50 (0)	1500 (5)	5	26
Pump Down 1	10	/	/	/	/	5	/
Stabilization 2	2	/	24	/	/	5	/
Passivation	5	/	24	50 (0)	1500 (3)	5	25
Pump Down 2	10	/	/	/	/	5	/
**Loop**							
Final $SF_{6}$	60	80	/	50 (0)	1500 (5)	5	26

**Table 2:** Sixty base DNA sequences as probe and target molecules and their molecular weights.

ssDNA	Sequence	Molecular Weight (kDa)
Probe	5${}^{\prime }$-NH${}_{2}$- TCA TCG GTC AGG TGC AAC AAA TTG ATA AGC	18
	AAT GCT TTT TTG GCC CTA TCT TCT AAC AGC-3${}^{\prime }$	
Target	5${}^{\prime }$-Biotin- GCT GTT AGA AGA TAG GGC CAA AAA AGC ATT	18
	GCT TAT CAA TTT GTT GCA CCT GAC CGA TGA-3${}^{\prime }$	

**Table 3:** Solutions used for the hybridization.

Solution	Components
Washing	2xSSC (Saline-Sodium Citrate)
PBS	1xPBS (Phosphate Buffered Saline Solution)
DNA	target ssDNA in 2xSSC, concentrations from 10 pm to 1 μm
MNP	streptavidin-coated MNP in PBS, ∼$10^{11}$ particles/mL

**Table 4:** DNA and magnetic nanoparticles hybridization protocol.

Step	Solution	Repetitions	Volume/Repetition μL	Flow μL/min	Step Pause min
1	Washing	1	>500	100	
2	DNA	1	200	100	
3	DNA	1	300	20	>5
4	Washing	1	300	100	>5
5	PBS	1	300	100	>5
6	MNP	1	200	100	
7	MNP	4	25	20	>4
8	PBS	1	>500	20	
				Total time:	>100 min

## Data Availability

Experimental data are available upon request.
